# The Effect of Fibre-Rich Gluten Free Products on Glycaemic Regulation, Gut Symptoms, and Targeted Gut Microbial Taxa in Women with Coeliac Disease: A Randomised Controlled Trial

**DOI:** 10.3390/nu18213462

**Published:** 2026-10-22

**Authors:** Solveig I. N. Watters, Mari C. W. Myhrstad, Elisabeth Austad, Line Gaundal, Henrik M. Roager, Terje F. Gjøvaag, Monica J. Hellmann, Lisa Garnweidner-Holme, Lise Friis Pedersen, Knut E. A. Lundin, Christine Henriksen, Vibeke H. Telle-Hansen

**Affiliations:** 1 Department of Nursing and Health Promotion, Faculty of Health Sciences, Oslo Metropolitan University, St. Olavs Plass, P.O. Box 4, 0130 Oslo, Norway; 2 Department of Nutrition, Exercise and Sports, University of Copenhagen, Rolighedvej 26, 1958 Frederiksberg, Denmark; 3 Department of Rehabilitation Science and Health Technology, Faculty of Health Sciences, Oslo Metropolitan University, 0130 Oslo, Norway; 4 Det Glutenfrie Verksted, Nordseterveien 26A, 1176 Oslo, Norway; 5 Norwegian Coeliac Society, 0158 Oslo, Norway; 6 Institute of Clinical Medicine, Faculty of Medicine, University of Oslo, Blindern, P.O. Box 1171, 0318 Oslo, Norway; knut.lundin@medisin.uio.no; 7 Department of Gastroenterology, Oslo University Hospital, Rikshospitalet, 0424 Oslo, Norway; 8 Department of Nutrition, Institute of Basic Medical Sciences, Faculty of Medicine, University of Oslo, Blindern, P.O. Box 1072, 0317 Oslo, Norway

**Keywords:** gluten-free products, pseudocereals, dietary fibre, glycaemic regulation, gut symptoms, gut microbiota, coeliac disease, randomised controlled trial

## Abstract

**Background***:* The only treatment for coeliac disease (CeD) is a gluten-free diet. Gluten-containing cereals constitute a major source of dietary fibre, raising concerns about fibre intake for individuals with CeD. **Objective**: The present study aimed to investigate the effect of pseudocereal-based, fibre-rich gluten-free products on glycaemic regulation in women with CeD. Secondary endpoints were changes in gut symptoms, targeted gut microbial taxa, and inflammatory and metabolic markers. **Methods**: This double-blind, randomised, controlled trial was conducted remotely and included 30 women with CeD. Participants were allocated to consume either pseudocereal-based, fibre-rich gluten-free products (experimental) or benchmark products (control) for three weeks. Before and after the interventions, participants underwent an oral glucose tolerance test (OGTT), reported gastrointestinal symptoms, and provided blood samples to analyse micronutrients, metabolic markers, and a satiety hormone, and faecal samples for targeted gut microbial taxa analyses. **Results**: There was no significant difference in the glycaemic response after the intake of fibre-rich products compared with control products. However, a significant reduction in glucose iAUC was observed within the experimental group (*p* = 0.049). There was no significant change in gut symptoms within or between groups, except for a reduction in diarrhoea in the control group compared with the experimental group. The abundance of five bacterial species was significantly decreased after intake of control products compared to experimental products. **Conclusions**: Pseudocereal-based, fibre-rich gluten-free products may be included in the diet for women with CeD without affecting gastrointestinal symptoms. Whether these fibre-rich products improve glycaemic regulation and exert effects via alterations in the gut microbiota needs to be further investigated.

## 1. Introduction

Gluten-related disorders, such as coeliac disease (CeD), affect millions of individuals worldwide, with a global prevalence between 1% and 1.4% [1,2]. Once considered rare, CeD is now recognised as one of the most common lifelong diseases worldwide and a major public health challenge [1,3]. CeD is a chronic, immune-mediated disease characterised by inflammation and villous atrophy of the small intestine, triggered by the ingestion of gluten proteins in wheat (gliadin and glutenin), rye (secalin), and barley (hordein) [4,5,6]. Despite the advances in understanding disease pathology, a gluten-free diet remains the only effective treatment for CeD [5]. Strict adherence to the gluten-free diet is critical in order to achieve clinical and histological remission and reduce the risk of developing long-term health complications [7,8]. However, this dietary regimen entails the elimination of gluten-containing foods such as bread and breakfast cereals, which represent staple sources of dietary fibre in most European households [9]. Gluten-free products often replace their gluten-containing counterparts and make up a significant part of gluten-free diets [10]. In contrast to their gluten-containing equivalents, gluten-free products typically contain less dietary fibre and protein and more salt, sugar, and saturated fat [10,11,12,13]. Consequently, adults adhering to such a diet, in particular individuals with CeD, continue to fall short of the recommended dietary intake [14].

Several non-gluten cereals and starches (e.g., rice, maize, sorghum, and potato/pea starch) are commonly used as a substitute for wheat flour in gluten-free products despite their low fibre content [15,16]. Hence, the widespread use of gluten-free products low in fibre may contribute to the inadequate fibre intakes observed within habitual gluten-free diets [14]. Incorporating oats and pseudocereals into gluten-free products could increase the fibre content and, overall, improve the nutritional quality. Oats are considered gluten-free, provided they are uncontaminated by wheat, barley, or rye during production [15,17]. The primary fibre in oats is β-glucan, which has several potential physiological benefits, including a reduced postprandial glycaemic response, as recognised by the European Food Safety Authority (EFSA) [18]. Emerging evidence also suggests that certain pseudocereals may offer health benefits similar to those of β-glucan [19,20,21,22,23,24,25,26]. Pseudocereals are edible fruits or seeds of non-grass species consumed similarly to cereals [27,28]. In comparison to rice-, maize-, and starch-based products, pseudocereal flours generally provide a higher fibre content, making them suitable candidates for gluten-free products. Examples include buckwheat, amaranth, and quinoa, which are characterised by the absence of gluten proteins [15,29]. In addition to fibre, pseudocereals provide high-quality protein and micronutrients (e.g., iron, selenium, and zinc), thereby improving the overall nutritional quality of the gluten-free products [30]. Notably, buckwheat and quinoa have been shown to lower postprandial glycaemia, suggesting these crops could be a valuable source of fibre in gluten-free products [23,24,31,32,33,34,35,36]. Nevertheless, exchanging gluten-containing flour with pseudocereal flour in traditional cereal-based products presents technical challenges, such as a less favourable texture, colour, and taste [37,38]. While many non-gluten cereals and starches are relatively neutral in flavour, pseudocereals can be perceived as somewhat bitter due to their high content of phenolic substances [39]. Developing palatable, high-fibre gluten-free products using pseudocereals exclusively is, therefore, challenging. However, combining pseudocereals in lower percentages with other gluten-free flours, such as oats, can improve flavour perception and increase the fibre content of gluten-free products [37]. Notably, recent findings suggest individuals with CeD may experience impaired microbial fibre metabolism, and certain dietary fibres might trigger gastrointestinal symptoms [40]. Consequently, it remains unclear whether pseudocereals are well-tolerated by these individuals or if they could exacerbate gastrointestinal issues. Further research is needed to establish their safe use for increasing fibre in gluten-free products.

As the gluten-free diet is a lifelong therapy in CeD, increasing the dietary fibre content and, overall, improving the nutritional profile of gluten-free products can represent an important strategy in supporting long-term health, particularly considering substantial evidence linking a higher fibre intake with improved overall metabolic health [41,42]. The beneficial effects of dietary fibres have been attributed to key pathways, including glucose and lipid regulation and insulin sensitivity [41,43,44,45,46]. Additionally, fibres appear to play a significant role in modulating the composition, diversity, and functionality of the gut microbiota and seem to be a mediator of the health-beneficial effects they elicit [47]. Hence, the long-term adherence to a gluten-free diet low in dietary fibres may pose a risk for health complications. One systematic review and meta-analysis found that the gluten-free diet has a beneficial effect on cardiometabolic risk factors and no significant effect on glucose metabolism [48]. In contrast, two other systematic reviews, one of which included a meta-analysis, indicated the gluten-free diet may increase risk factors such as fasting glycaemia, total cholesterol, and body mass index (BMI) [49,50].

Considering the low dietary fibre content of many commonly used gluten-free products, pseudocereal-based gluten-free products warrant an evaluation for their effects on glycaemic regulation, gut symptoms, and gut microbiota. We hypothesise that pseudocereal-based products high in fibre will improve glycaemic regulation, negatively affect gut symptoms, and beneficially influence metabolic markers and gut microbial species in people with CeD. Accordingly, the main aim of the present study was to investigate the effect of pseudocereal-based, gluten-free products rich in fibre on glycaemic regulation, and the secondary aims were to investigate the effects on gut symptoms, metabolic- and inflammatory markers, and gut microbiota, targeting a panel of 108 species in people with CeD.

## 2. Experimental Methods

### 2.1. Regulatory Approval

The study was conducted according to the guidelines in the Declaration of Helsinki, and all procedures involving human participants were approved by the Regional Committees for Medical and Health Research Ethics Sør-Øst (#83004) and Norwegian Service of Research Data (#437313). The study was registered at Clinical Trials (https://clinicaltrials.gov/), registration identification number: NCT05135923.

### 2.2. Participants

**Figure 1:**
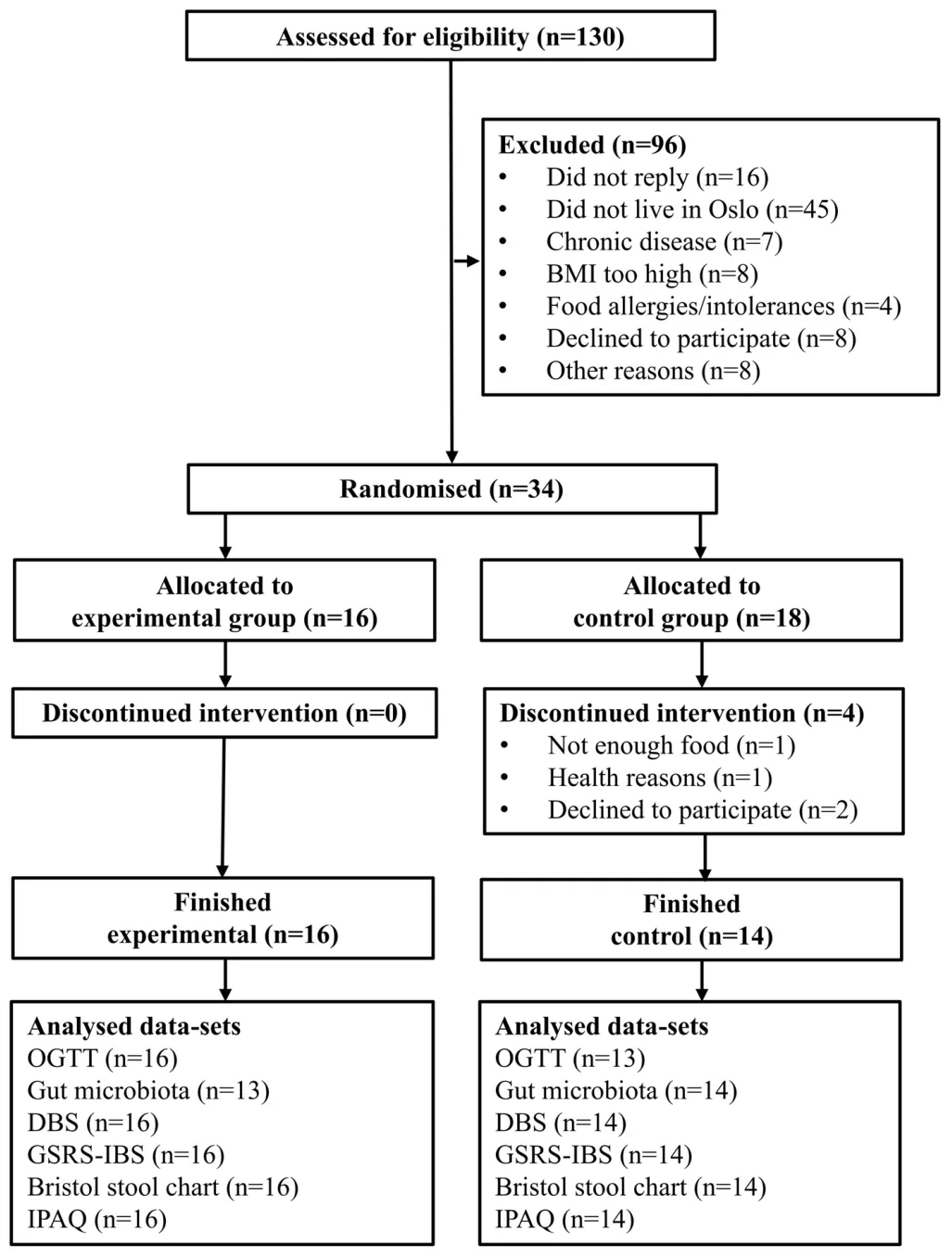
Flow chart of the participants. Abbreviations: BMI: Body Mass Index.

Individuals diagnosed with CeD were recruited between September and November 2021 through the Oslo Metropolitan University (OsloMet) website and social media (Facebook and Instagram) to participate in this remotely conducted double-blind, randomised, controlled pilot study. In total, 130 individuals were invited to participate, and 56 individuals were screened to recruit 30 participants for the study. Participants were screened for eligibility according to the following inclusion and exclusion criteria. Participants qualified to participate if they met all the criteria and provided written informed consent. Further descriptions of the participants lost during follow-up are outlined in the flow chart (Figure 1).

Men and women between the ages of 18 and 65 years, normal-weight (BMI > 18.5 kg/m^2^) or slightly overweight (BMI ≤ 27 kg/m^2^), diagnosed with CeD were considered for the study. The CeD diagnosis must have been confirmed by a medical practitioner through serology or biopsy and not self-diagnosed [7]. The average body mass index in the Norwegian population is 26.5 kg/m^2^ for men and 25.6 kg/m^2^ for women. Therefore, the BMI inclusion criterion includes participants with a BMI of less than 27 kg/m^2^. The exclusion criteria were as follows: any food allergies (except for CeD), intolerances, or chronic metabolic diseases (e.g., diabetes, cardiovascular disease, and cancer); medication (except oral contraception) and intestinal diseases (e.g., inflammatory bowel disease and irritable bowel syndrome); antibiotic treatment within the previous three months and during the study; pregnancy or breastfeeding; planned weight reduction (less than 5% weight change); tobacco use; and high alcohol consumption (>40 g/day).

### 2.3. Pilot Study Design

The pilot study employed a randomised, double-blind, controlled, parallel design. All interaction and communication with the participants were managed remotely by e-mail, phone, and e-visitation, following restrictions related to the COVID-19 pandemic. The screening process was conducted by e-visitation and consisted of a health examination, including recording of pre-existing medical history and self-reported height and weight, calculation of BMI, and signing of the informed consent form. The participants also received and completed a validated food and frequency questionnaire (FFQ) designed to assess nutrient and food intake during the past year [51]. Average daily intake of energy and nutrients was calculated using the food database KBS system developed at the Department of Nutrition, University of Oslo, Norway [52]. Additionally, data collection kits and instructions were demonstrated, allowing participants to get acquainted with the collection methods.

**Figure 2:**
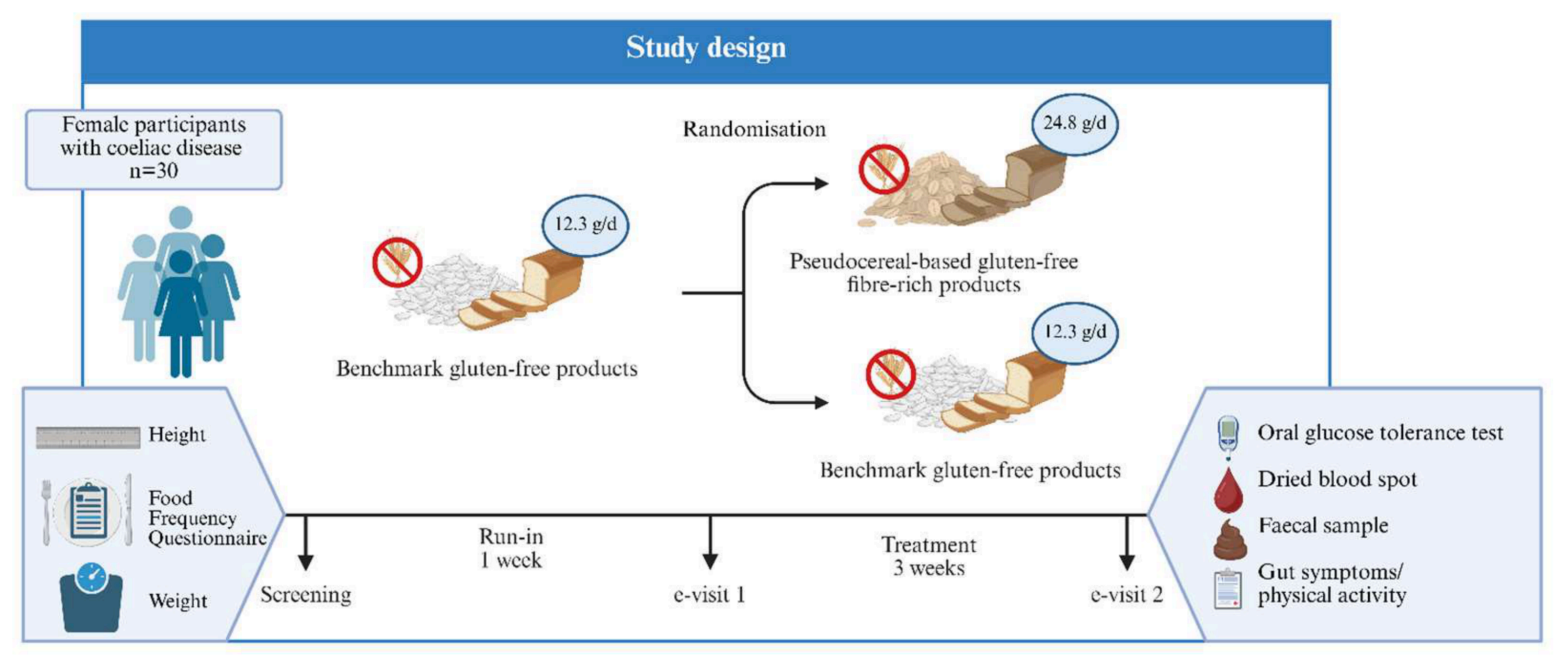
Study design of the double-blind, randomised, controlled study in which thirty volunteers with coeliac disease consumed experimental products (fibre-rich gluten-free, n = 16) or control products (benchmark gluten-free, n = 14) for three weeks. The participants consumed control products during the run-in. An oral glucose tolerance test (0–120 min) was performed at each e-visit, and glycaemic response was measured and fasted blood samples were collected and analysed for metabolic- and inflammatory markers and satiety hormones. At each e-visit, faecal samples were collected and analysed for gut microbiota, and gut symptoms (Gastrointestinal Symptoms Rating Scale-IBS, and Bristol Stool Chart) were also assessed. In addition, the participants completed a food frequency questionnaire (FFQ) during screening, and anthropometric measurements were collected during screening and at each e-visit.

Participants then commenced the run-in period, during which they received control products for one week (Figure 2). Following the run-in period, the participants were randomly allocated into two groups, receiving either optimised fibre-rich gluten-free products (experimental) or benchmark products (control) for three weeks (Figure 2). The study had a total duration of four weeks. Prior to the run-in period and each intervention week (four time points), the participants were delivered study products (control or experimental). During the first delivery, the participants also received the FFQ, and during the second and fourth deliveries, they also received data collection kits and ready-made return envelopes. The kits included materials for collecting faecal microbiota and dried blood spots (DBS), a blood glucose monitoring system, glucose solution for an oral glucose tolerance test (OGTT), and written instructions for collection. Throughout the study, the participants attended three e-visits (screening, e-visit 1 and e-visit 2) (Figure 2). E-visit 1 was conducted at the start of the intervention period and e-visit 2 at the end (Figure 2). During e-visits 1 and 2, participants collected data according to instructions and under the supervision of a researcher. On the morning of each e-visit, the participants collected fasted (≥12 h) DBS samples and then underwent an OGTT, while faecal samples were collected the previous day. The blood and faecal samples were returned to the study site (OsloMet, campus Kjeller) by regular mail in ready-made envelopes after each e-visit. Additionally, participants reported gastrointestinal symptoms and physical activity levels by responding to three online questionnaires, Gastrointestinal Symptom Rating Scale-Irritable Bowel Syndrome (GSRS-IBS), Bristol Stool Chart (BSC), and IPAQ-Short Form (IPAQ-SF), which they received by e-mail, and answered a case report form (CRF) detailing adherence to the study protocol, diet, and any changes in health status. The first five participants from each intervention group, ten in total, to complete the study were offered individual interviews to share their experiences with consuming gluten-free bread, which has been published elsewhere [53].

### 2.4. Study Intervention

The present study included fibre-rich gluten-free products (experimental) or benchmark gluten-free products (control). The experimental products consisted of bread, bread rolls, and muesli with chia, developed by Det Glutenfrie Verkstedet (DGV, Oslo, Norway) in collaboration with the project group during spring 2021. The recipes were optimised with oats and pseudocereals (e.g., buckwheat and quinoa) developed to meet the requirement for the Nordic Keyhole label [54] and the EFSA nutrition claim “rich in fibre” (minimum 6 g fibre/100 g product [55]. One week’s supply of experimental products included two breads (800 g each bread), seven rolls (50 g each roll), and one bag of muesli (420 g). The participants were instructed to consume a minimum of four slices of bread (approx. 50 g each slice), one roll (approx. 50 g), and one portion of muesli (60 g) daily, providing 24.8 g fibre/day. The control products consisted of two types of bread, gluten-free grain bread (Glutenfri Kornbrød) and gluten-free oat bread (Glutenfri Havrebrød) from Brisk bakery (Ålesund, Norway) and corn flakes from DGV. These were commercially available gluten-free products, including oat-containing products, selected to reflect real-world dietary practice in CeD. One week’s supply of the control products included four breads (500 g each of bread) and two bags of cornflakes (200 g per bag). The participants were instructed to consume a minimum of six slices of bread (approx. 35 g of each slice) and one portion of cornflakes (60 g) daily, providing 12.3 g fibre/day. The participants registered compliance by recording the number of slices and rolls, and the amount of cornflakes/muesli consumed during the intervention weeks. Compliance with the protocol was monitored continuously at the end of each study week via e-mail. Participants who were unable to consume the minimum amount were instructed to withdraw from the study. All participants complied with the minimum amounts set in the protocol. The nutritional composition of the experimental and control products was analysed by Eurofins (Eurofins Food & Feed Testing Norway AS, Ålesund, Norway). Participants were required to replace all bread and cereals in their regular diet with the gluten-free products provided in the study. Participants were to refrain from making any further changes to their diet, physical activity, and overall lifestyle during the study period, except for the dietary substitutes provided by the study.

### 2.5. Randomisation and Blinding

After the run-in period, participants were assigned an ID number and randomly assigned following simple randomisation procedures to consume experimental or control products. The randomisation was conducted by a project member not involved in the data collection or analyses. The randomisation key was only accessed by the individuals who managed the randomisation and packaging of the study products. Additionally, the study products were packed in neutral packaging to ensure the participants were not informed of the type of products they received. Therefore, the study was double-blinded, but there is a chance that some participants recognised which products they received, as the benchmark products were already available for purchase in grocery shops. The codes for unblinding and treatment grouping were provided to the researcher responsible for the analyses when all statistical analyses were finalised. In this study, neither participants nor investigators were asked to guess the treatment allocation. However, participants were invited to take part in a qualitative interview at the end of the study period. Using a semi-structured interview guide, the participants were asked about the study breads. This article has been published [53].

### 2.6. Oral Glucose Tolerance Test

The participants underwent an OGTT at home during each e-visit, under the supervision of a researcher. The participants were provided with a glucose solution containing 82 g of glucose (D (+)-Glucose monohydrate), which is equivalent to 75 g of glucose, dissolved in 100 mL of water. The participants were instructed to consume the glucose solution within 10 min and then sample finger-prick capillary blood glucose using the Contour^®^Next blood glucose monitoring system. Capillary blood glucose was monitored at the following intervals: before consumption (fasting) and 15, 30, 60, 90, and 120 min after consuming the glucose solution. Throughout the OGTT, participants remained seated.

### 2.7. Faecal Sampling

The participants collected faecal samples the day before the e-visit to analyse gut microbiota. The participants received a package containing filter cards for faecal samples (Whatman^®^ 903 filter paper, a wooden lollipop stick applicator, an airtight specimen transfer bag containing a sachet of silica as desiccant material (EBF (Eastern Business Forms), Mauldin, SC, USA), a flushable toilet seat cover for stool collection (Fe-Col^®^, Col-group, Amsterdam, The Netherlands), and instructions on how to collect the faecal sample with the equipment provided. Participants were instructed to collect a faecal sample in the toilet seat cover, smear a thin layer of the sample on the filter card using the lollipop stick applicator, and let it air dry at ambient temperature for at least two hours before placing it in the foil bag. Samples were marked with the date and ID numbers and stored at room temperature until returned to the study site (OsloMet, campus Kjeller) by regular mail after each e-visit and stored at −80 °C at OsloMet, campus Kjeller, before being dispatched to Bio-Me (Bio-Me Microbiome Profiling, Oslo, Norway) for faecal microbiota analysis.

### 2.8. Analysis of Faecal Microbiota

Three discs of filter paper 6 mm in diameter were punched from each of the 59 faecal sampling cards. Microbial DNA was extracted using MagMAX™ Microbiome Ultra Nucleic Acid Isolation Kit (Thermo Fisher Scientific, Waltham, MA, USA). The bacterial cell wall was disrupted using Star Beater (VWR, West Chester, PA, USA) at 30 Hz for 2 min. Purified DNA was eluted in 200 μL MagMAX™ Elution Buffer, and DNA was quantified using the Quant-iT™ PicoGreen™ dsDNA Reagent (Thermo Fisher Scientific, Waltham, MA, USA) and an Infinite F200 Fluorescence Microplate Reader (Tecan Group AG, Männedorf, Switzerland). DNA was analysed using the Precision Microbiome Profiling (PMP™, Bio-Me, Oslo, Norway), consisting of custom-made OpenArray^®^ qPCR panels amplifying 107 measured bacterial and archaeal species/subspecies and 1 fungal genus (Candida) (108 species in total) (TaqMan^®^ OpenArray^®^ Real-Time PCR Plates Protocol), documented to be prevalent in the human gut microbiome and associated with disease or response to dietary treatments. Microbial taxa were quantified in absolute genomic copies per μL, interpolated from standard curves based on quantified reference isolates from DSMZ (Leibniz Institute, Braunschweig, Germany) or ATCC (American Type Culture Collection, Manassas, VA, USA). To enable comparison between samples, normalised quantification (number of genomic copies per ng of DNA) was calculated by dividing the absolute quantification for each target by the total DNA concentration in a sample.

### 2.9. Blood Sampling

Fasted (>12 h) capillary blood samples were collected by DBS (Whatman^®^ 903 five spot blood card) to analyse Haemoglobin A1c (HbA1c), C-peptide, total cholesterol, and inflammatory markers (Interleukin (IL)-10, IL-17A, IL-6, IL1-β, IL-1Ra, IL-8, Tumor necrosis factor alpha (TNF-α), Interferon-gamma (IFN-γ), Monocyte chemoattractant protein-1 (MCP-1), and C-reactive protein (CRP)) [56,57]. The participants received a DBS kit and instructions on collecting the capillary blood samples with the provided equipment. The participants collected fasting (>12 h) capillary blood samples by finger-prick with a sterile lancet after sanitisation with an isopropyl alcohol swab. The participants discarded the first drop of blood before completing five spots with drops of capillary blood (250 μL) on two DBS cards. The cards were dried at ambient temperature for at least two hours before they were packed in an airtight specimen transfer bag containing a sachet of silica as desiccant material (Whatman^®^ foil barrier ziplock bag for 903 cards). The DBS samples were marked with the date and ID numbers and stored at room temperature until returned by regular mail to the study site (OsloMet, campus Kjeller) after each e-visit and stored at −80 °C before being dispatched to VITAS (Vitas Analytical Service, Oslo, Norway) for analysis.

### 2.10. Analysis of Blood Samples

HbA1c was quantified in whole blood and is given as mmol/mol. One DBS punch (3.1 mm) was haemolysed using 600 μL of lysate buffer. After 30 min of lysis at ambient temperature, 10 μL of lysate was treated with reagents 1 and 2 from Pointe Scientific (Medtest, Canton, MI, USA). The absorbance was read at 660 nm using Spark 10M microplate reader (Tecan, Männedorf, Switzerland). C-peptide was quantified by sandwich enzyme-linked immunosorbent assay (ELISA) and is given as pmol/L. Three DBS punches (3.2 mm) were punched into the kit plate with anti-C-peptide antibodies bound to the well. Assay buffer and Calibrator 0 were added, and C-peptide was extracted from the spots. The Ultrasensitive C-peptide kit (Mercodia AB, Uppsala, Sweden) manual was followed, and the absorbance was read spectrophotometrically at 450 nm using Spark 10M microplate reader (Tecan, Männedorf, Switzerland).

Total cholesterol was quantified using gas chromatography–flame ionisation detection (GC-FID) and is given as mmol/L. One DBS punch (4.7 mm) was methylated with sodium methylate. Cholesterol in whole blood is present as free cholesterol and cholesterol bonded to a fatty acid as cholesteryl ester. During sample preparation, a basic catalysed transesterification was performed, liberating free cholesterol from the cholesteryl ester and turning total cholesterol into its free form. After methylation (40 min, 450 revolutions per minute (rpm), 50 °C), 500 μL of internal standard C23 in hexane was added to 300 μL of distilled water. After mixing and centrifugation, 3 μL of the aliquot was injected into the GC-FID. GC-FID was performed with an Agilent 7890A Gas Chromatograph System (Agilent Technologies, Palo Alto, CA, USA). The fatty acid methyl esters and cholesterol were separated on a TR-FAME column (Thermo Scientific 30 m × 0.25 mm × 0.25 µm). Inflammatory markers (Interleukin-10, IL-17A, IL-6, IFN-γ, TNFα-, IL1-β, IL-1Ra, IL-8, MCP-1, and CRP) and PYY were quantified in DBS using the MSD Mesoscale Electrochemiluminescence (ECL) platform and are given as pg/mL. Two 3.1 mm punches were eluted in kit diluent for 2 h at 700 rpm at room temperature. The inflammatory markers were measured from the eluate using the MESO^®^ QuickPlex SQ 120 Multiplex Imager and the custom U-PLEX Biomarker Group 1 Multiplex assay (MSD, Rockville, MD, USA).

### 2.11. Measurements of Gastrointestinal Symptoms

Gastrointestinal symptoms were measured by the Gastrointestinal Symptoms Rating Scale-Irritable Bowel Syndrome (GSRS-IBS) and Bristol Stool Chart, distributed online by e-mail, and participants responded retrospectively during screening and each e-visit [58,59]. The participants rated themselves on the GSRS-IBS, consisting of 13 questions concerning gas, diarrhoea, constipation, and symptoms such as the urgent need to have a bowel movement, trouble emptying the bowel, and unusual satiety patterns, measuring abdominal symptoms. Each question in the questionnaire included a 7-point Likert scale ranging from no symptoms = “1 point”, minor (2 points) or mild discomfort (3 points), then moderate (4 points) and moderately severe discomfort (5 points), severe discomfort (6 points), and very severe discomfort = “7 points”, with a total score range of 13–91 points [58]. The GSRS-IBS comprises five sub-dimensions of gastrointestinal symptoms with respective score ranges: pain (2–14 points), bloating (3–21 points), constipation (2–14 points), diarrhoea (4–28 points), and satiety (2–14 points). The participants also classified their stools on the BSC [59]. The BSC classifies stool into 7 types, where types 1–2 are consistent with severe and mild constipation, 3–4 are normal, 5 is normal approaching diarrhoea, and 6–7 are consistent with diarrhoea. All answers were collected and stored in Services for Sensitive Data (Tjenester for sensitive data: TSD).

### 2.12. Physical Activity

The participants received the link to the IPAQ-SF by e-mail and were asked to complete it during each e-visit to determine their habitual physical activity levels [60]. The short form of the IPAQ is a physical activity recall for the last seven days. The participants were asked to recall minutes, hours, and days spent sitting, walking, and doing moderate and vigorous physical activity. All answers were collected and stored in Services for Sensitive Data (Tjenester for sensitive data: TSD).

### 2.13. Statistical Analysis

The primary outcome measures, as described in Clinical Trials (NCT05135923), are fasting and postprandial blood glucose response after an OGTT. Secondary endpoints included changes in metabolic markers, inflammatory markers, PYY, gut microbiota, and gastrointestinal symptoms. Given the exploratory nature of this pilot study, power calculations were not performed.

Of the 30 participants in the study, 27 collected stool samples, 29 underwent OGTT, and all 30 collected DBS and reported gastrointestinal symptoms. Metabolic markers, inflammatory markers, PYY, and gut microbial species not detectable in more than 50% of participants at each time point were excluded from the statistical analyses. Subsequently, 5 inflammatory markers (IL-10, IL-17A, IL-6, IFN-γ, and TNF-α) and 25 gut microbiota species were excluded from the statistical analysis. All the metabolic markers, PYY, 4 of the 9 inflammatory markers (IL1-β, IL-1Ra, IL-8, and MCP-1), 83 of the 108 gut microbiota species, and gut symptoms were subject to statistical analysis. Missing values in the analysed data set were extrapolated from all the participants’ mean values at that time point. At e-visit 1, two participants in the control group failed to complete the OGTT and received estimated values. Additionally, a participant in the control group provided a DBS sample unsuitable for analysis, and another participant provided a DBS sample unsuitable for PYY measurement. Therefore, estimated values were assigned to both. Two participants did not submit the GSRS-IBS at e-visit 1 and were assigned estimated values. For markers with concentrations lower than the lower limit of quantitation (LLOQ), we assigned the respective LLOQ divided by two (LLOQ/2) for the given marker. In the control group, two values for CRP, IL-1β, and IL-1Ra were replaced with LLOQ/2. In the experimental group, one value for CRP and IL-8 and two and four values for IL-1β and IL-1Ra were replaced with LLOQ/2, respectively.

In the statistical analysis of the gut microbiota, microbial alpha diversity was quantified using the Shannon index based on the normalised quantification of bacterial taxa. Non-parametric tests were used to assess differences in Shannon index between interventions (Mann–Whitney U test for experimental vs. control) and within interventions (Wilcoxon signed-rank test for e-visit 2 vs. e-visit 1). For species-level analysis, the normalised quantification counts were logarithmically transformed using base 2 (Log2) to reduce skewness in the original data. The effects of the intervention were quantified as log2 ratios for each participant, calculated as the difference between log2-transformed values at e-visit 2 and e-visit 1. Further, parametric tests were used to assess the difference between interventions (independent *t*-test) (experimental vs. control) and within interventions (paired *t*-test) (e-visit 1 vs. e-visit 2) based on the log2 ratio. Mean values, standard deviation (SD) and 95% confidence intervals are shown. To correct for multiple testing across the 83 bacterial taxa, we adjusted *p*-values using the Benjamini–Hochberg False Discovery Rate (FDR) method. Adjustments were performed in Microsoft Excel, and corresponding q-values are presented. The blood glucose load (incremental area under the curve (iAUC)) for each participant was calculated by subtracting the fasting value (0 min) from the corresponding values 15, 30, 60, 90, and 120 min after consuming the glucose solution, and then calculated using the trapezoidal rule (*A* = (*y*1 + *y*2) × (*x*2 − *x*1)/2). For the statistical analyses of fasting blood glucose, iAUC, metabolic markers, inflammatory markers, and gut symptoms, non-parametric tests were used to assess differences between interventions (the Mann–Whitney U test) (experimental vs. control) and within interventions (the Wilcoxon signed-rank test) (e-visit 2 vs. e-visit 1). Median values and 25–75 percentiles are shown. The significance level was set to 5% (*p* ≤ 0.05). The data were processed in Microsoft 365 MSO (version 2302) and statistically analysed in IBM SPSS Statistics (version 28). All figures were designed using GraphPad Prism 11 (version 11.0.2).

## 3. Results

### 3.1. Baseline Characteristics

Thirty participants (all females) completed this randomised, controlled, double-blind study, and the baseline characteristics of the participants are shown in Table 1. The participants were normal-weight (median BMI of 22.4 kg/m^2^) adults (median age of 31 years) with fasting glucose, C-peptide, total cholesterol, and HbA1c levels within the normal range (Table 1).

### 3.2. Habitual Diet

The participants’ habitual diet was determined at screening before starting the interventions by FFQ and is shown in Table 2 and Table 3. The median daily intake of energy, fat, protein, and carbohydrates in the total study population was 2164 kcal, 36.9 E%, 16.8 E%, and 41.4 E%, respectively (Table 2). The participants had a daily median intake of 30.5 g of fibre, 120.3 g of bread, and 142.9 g of cereals at baseline. Prior to the intervention, there were no significant differences in total energy intake, macronutrients, or fibre (Table 2), or in the intake of key fibre sources, including bread and cereal, between the groups (Table 3).

### 3.3. The Study Products

The nutritional composition of the experimental and control products is presented in Table 4. The daily intake of fibre provided by the experimental products was twice as high, at 24.8 g, compared with 12.3 g from the control products. The experimental products provided a higher daily intake of energy, protein, and fat (882 kcal, 33.9 g, and 39.6 g, respectively) and a lower carbohydrate intake (85.3 g) compared to the control products (773 kcal, 9.3 g, 13.2 g, and 146.3 g, respectively). In addition, the experimental products provided more saturated fat, monounsaturated fatty acids, and polyunsaturated fatty acids (5.6 g, 12.5 g, and 19.6 g, respectively) compared with the control products (1.5 g, 6.2 g, and 4.8 g, respectively) (Table 4).

### 3.4. Glycaemic Regulation

**Figure 3:**
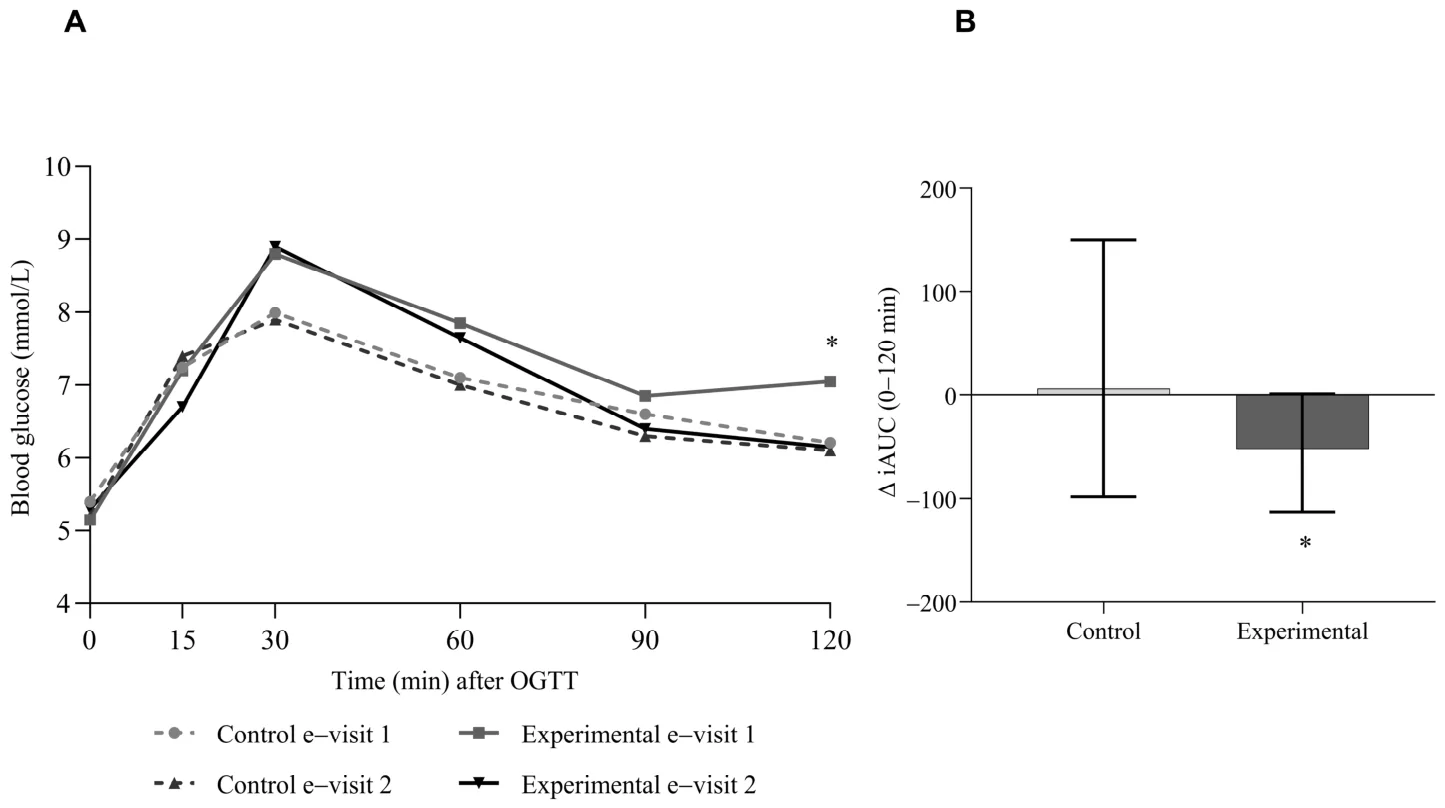
Postprandial blood glucose response after intake of experimental products (fibre-rich gluten-free, n = 16) or control products (benchmark gluten-free, n = 13). The postprandial blood glucose response from 0–120 min at each e-visit (**A**) and the change in incremental area under the curve during intervention (**B**) are shown after intake of both experimental and control products. Data are given as median (25th–75th percentiles). Differences in change between groups were analysed by the Mann–Whitney U test, and differences within groups were analysed by the Wilcoxon signed-rank test. Significant differences (*p* ≤ 0.05) are indicated with *. Abbreviations: iAUC: Incremental Area Under the Curve; OGTT: Oral Glucose Tolerance Test.

**Figure 4:**
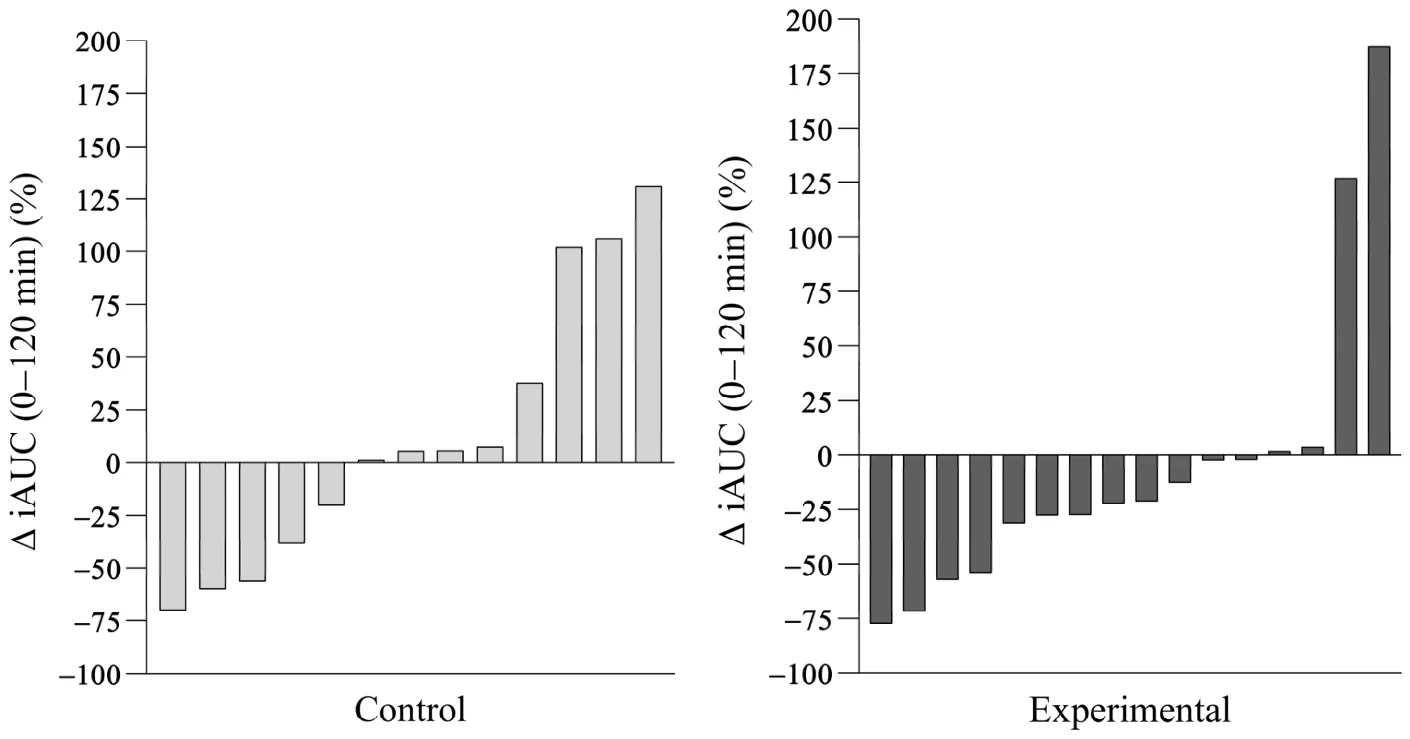
Individual changes in glycaemic response. Each bar represents the individual percentage change in iAUC (0–120 min) after a three-week intervention with control products (benchmark gluten-free) or experimental products (fibre-rich gluten-free). Abbreviations: iAUC: incremental area under the curve.

An OGTT was performed before and after the interventions to assess the effects of fibre-rich gluten-free products compared to control products on glycaemic regulation. No significant difference in the change in glucose iAUC was observed between groups (Figure 3B). However, there was a significant reduction in glucose iAUC within the experimental group (*p* = 0.049) (Figure 3B). We found that the change in blood glucose level at 120 min after the OGTT was significantly different and decreased after consuming the experimental products compared to the control products (*p* = 0.04). Within the experimental group, the glucose level at 120 min was significantly reduced compared to before the intervention (*p* = 0.02) (Figure 3A). Furthermore, a reduction in the postprandial glucose response (iAUC) was shown in 12 participants after the intake of the experimental products, while four participants had an increased response. After the intake of the control products, eight participants increased while five decreased their postprandial glucose response (Figure 4).

### 3.5. Gastrointestinal Symptoms

The change in the GSRS-IBS diarrhoea score was significantly greater in the control group compared to the experimental group (*p* = 0.04) (Table 5). Within the control group, the GSRS-IBS diarrhoea score was significantly reduced (*p* = 0.01). No other significant changes (increase or decrease) were observed in any of the GSRS-IBS symptoms after the intake of fibre-rich experimental products. Additionally, there were no significant changes in the BSC scores after consuming the experimental products (0.00 (−0.50–0.63)) compared with the control products (0.50 (0.00–1.00)) (Table 5), nor within either group.

### 3.6. Gut Microbiota

To investigate changes in species richness and evenness after the interventions, the Shannon diversity index was calculated. No significant changes in the Shannon index were observed between groups or within groups (Table 6). At the species level, the change in abundance of 5 microbial species (out of 83) was significantly decreased after the intake of control products compared with experimental products. Specifically, *Anaerostipes hadrus* (*p* = 0.02), *Bacteroides caccae* (*p* = 0.04), *Dorea longicatena* (*p* = 0.03), *Escherichia coli* (*p* = 0.02), and *Faecalibacterium prausnitzii A2-165* (*p* = 0.05) were significantly decreased within the control group (Table 6). However, none of these differences remained statistically significant after an adjustment for multiple testing using the Benjamini–Hochberg False Discovery Rate (FDR), with all corresponding q-values exceeding 0.05 (Table 6). No significant changes in the gut microbiota composition were observed within the experimental group. Within the control group, the abundance of eight bacterial species and one fungal genus (Candida) significantly decreased after the intervention: *Anaerostipes hadrus* (*p* = 0.03), *Bacteroides caccae* (*p* = 0.02), *Coprococcus catus* (*p* = 0.03), *Dorea longicatena* (*p* = 0.03), *Escherichia coli* (*p* = 0.03), *Faecalibacterium prausnitzii A2-165* (*p* = 0.02), *Phascolarctobacterium faecium* (*p* = 0.02), *Ruminococcus lactaris* (*p* = 0.03), and *Candida* (genus) (*p* = 0.02). The complete list of all bacterial species analysed in this study is provided in Supplementary Table S1.

### 3.7. Metabolic Markers and Inflammatory Markers

Next, we investigated whether the consumption of the experimental products influenced metabolic- and inflammatory markers compared with the control products. There were no significant differences in physical activity, metabolic markers (total cholesterol and C-peptide), inflammatory markers (IL-1β, IL-1Ra, IL-8, and MCP-1) or PYY between or within the intervention groups (Table 5).

## 4. Discussion

We investigated the effect of pseudocereal-based fibre-rich products on glycaemic regulation, gut symptoms, metabolic- and inflammatory markers, and gut microbiota, compared with benchmark control products in women with CeD. No significant difference in glucose iAUC was observed between groups. However, a significant decrease in iAUC was observed within the experimental group. Additionally, the change in blood glucose level at 120 min after the OGTT was significantly different between groups and decreased after consuming experimental products. There was no significant increase in gut symptoms between or within groups, except for a reduction in diarrhoea in the control group compared with the experimental group. The change in abundance of five gut bacterial species was significantly different, showing a greater reduction following consumption of control products compared with the experimental products.

The fibre provided by the experimental products was 24.8 g/day, twice that of the 12.3 g/day provided by the control products. The beneficial effect of fibre-rich foods on the blood glucose response is well documented in healthy subjects [41,61], and we would have expected to observe differences in glycaemic regulation between the two groups. The study was not fully controlled, and we do not know the total fibre intake of the participants. The lack of effect could therefore be ascribed to an insufficient difference in the fibre intake between the groups or to the control products also containing some oats. Oats are high in β-glucan, a soluble fibre demonstrated to reduce postprandial blood glucose responses [62,63]. Although no significantly different changes were detected between the groups, the individual glycaemic responses showed reduced iAUC levels in 75% (12 of 16) of the participants in the experimental group, whereas this was observed for only 39% (5 out of 13) of the participants in the control group. Although the reductions observed in the experimental group are noteworthy and may indicate a potential benefit, the difference between groups did not reach statistical significance. In addition to oats, buckwheat and quinoa were the primary sources of fibre in the experimental products. While it has been suggested that these pseudocereals may improve glycaemic regulation [23,24,32,64], several studies have failed to find any glucose-lowering effects when consuming buckwheat and quinoa [22,25,26,36,65,66,67]. Soluble fibre is considered the primary contributor to the glucose-lowering effects of dietary fibres due to its beneficial viscous properties, whereas insoluble fibre tends to accelerate small intestine transit and regulate bowel movements [68,69]. The current study analysed only the total fibre content. Thus, the specific amounts of soluble and insoluble fibre in the study products remain unknown. Nonetheless, previous studies have shown that buckwheat and quinoa primarily contain insoluble fibre and, only to a lesser extent, soluble fibre [29]. Taken together, although the experimental products contained more fibre than the control products, the amount of soluble fibre may not have been sufficient to induce significant glucose-lowering effects compared with the control products. Furthermore, whether fibre from buckwheat and quinoa may have contributed to the glucose reduction observed within the experimental group remains unclear.

Conventional gluten-free products are typically starch-based and low in nutrients. This study investigated the impact of replacing commercially available gluten-free products with pseudocereal-based alternatives that are naturally richer in nutrients. Pseudocereals not only increase the fibre content but also provide higher levels of protein and fat. Adjusting the products to match all macronutrients would have required the use of isolated fibre or extensive food processing, compromising the objective of the study. Despite a substantial additional fibre contribution from the experimental products (24.8 g/day) comparedwitho the control products (12.3 g/day), gut symptoms did not significantly increase within the experimental group (Table 5). Both groups showed a reduction in diarrhoea after the intervention, although this change was significantly larger in the control group compared to the experimental group. While one of the known effects of fibre intake is the effect on gut motility and prevention of constipation, excessive intake may lead to the aggravation of gastrointestinal symptoms [68,70]. This was not observed in the present study. These findings suggest that pseudocereals can be incorporated into gluten-free products to enhance dietary fibre and overall nutritional quality without compromising gastrointestinal symptoms.

During the last decade, the effects of diet and gut microbiota on metabolic regulation have been extensively investigated, aiming for better prevention and/or treatment of various diseases. As the present study investigated a higher fibre intake in people with CeD, the microbiota was analysed as a secondary endpoint, aiming to generate new hypotheses. Of the 83 microbial species included in the analysis, the change in abundance of *Anaerostipes hadrus*, *Bacteroides caccae*, *Dorea longicatena*, *Escherichia coli*, and *Faecalibacterium prausnitzii A2-165* was significantly decreased following intake of the control products compared with the experimental products. However, these differences did not remain statistically significant after adjusting for multiple testing (Table 6). The abundance of the genus *Faecalibacterium* and the species *F. prausnitzii* has previously been shown to be significantly reduced in adults with CeD compared with healthy individuals [71,72]. *F*. *prausnitzii* is known to degrade dietary fibre and produce butyrate, which could exert a protective role in the mucosa of healthy individuals [73], and reduced levels of this species have been shown to be associated with inflammation [74]. Whether the decrease in *F. prausnitzii* abundance following consumption of the control products could impact health needs to be further investigated. We found that the change in *E. coli* abundance was significantly different between the groups, characterised by a reduction following intake of control products compared with experimental products. The abundance of *E. coli* has previously been demonstrated to be higher in individuals with untreated CeD [75] and detected more often in individuals with CeD than in healthy controls [76]. Besides containing several distinct species that can cause various diseases, most *E. coli* species in the human gut are commensals [77]. Therefore, it remains to be established whether these changes in *E. coli* abundance would have any health implications.

The gut microbiota in individuals with CeD is different from that of individuals without CeD [78]. It is currently unknown whether these differences may affect the gut microbiota’s response to dietary changes, particularly following an increased fibre intake [79,80]. Despite the high fibre content in the experimental products, we did not observe any changes in the gut microbiota after the experimental intervention. Further research is therefore warranted in order to investigate whether the gut microbiota of individuals with CeD may cause an alteration in the microbiome response to dietary fibre. Before entering the study, the participants had a relatively high fibre intake of 30.5 g/day. It is therefore possible that the intake of the control products caused a decrease in total fibre intake, whereas the experimental products maintained the total fibre content at a similar level compared with their habitual diet. In addition, the lack of effects on the gut microbiota after the intake of experimental products may be attributed to the specific types of fibres present in these products. Whereas oats contain a substantial amount of soluble fibre [81], buckwheat and quinoa are reported to have a higher content of insoluble fibre [29]. While the gut microbiota can ferment and utilise both soluble and insoluble fibre, soluble fibre is generally recognised as being more easily fermentable by the gut microbiota [82]. Therefore, the lack of changes in the gut microbiota after the intake of fibre-rich gluten-free products may also be due to the fact that the products predominantly contained insoluble fibre. In the present study, we used a targeted approach to the gut microbiota analyses, and the panel included the most common gut bacterial species found in humans [83]. The gut microbiota results did not remain statistically significant after an adjustment for multiple testing (Table 6); these findings may guide future studies and explore how optimised gluten-free products could affect certain gut bacteria.

## 5. Limitations

This remotely conducted pilot study is exploratory by nature and was performed to generate new hypotheses. Due to the short study period and small sample size, the use of a parallel design presents a limitation. Additionally, mean-value imputation was employed to address the missing data. While this method maintained the sample size, it may have introduced minor bias to the data distribution or interpretation of the results. One advantage of conducting studies remotely is the ability to reach new participants through expanded geographic access and flexibility to accommodate time-poor schedules for interviews [84]. The remote sample collection proved to be more challenging than anticipated. Although participants collected data following instructions and were supervised by a researcher, some were unsuccessful in collecting samples, and certain samples were unsuitable for analysis. Thus, five inflammatory markers were excluded due to sample collection or quality issues, reducing the statistical power to detect inflammatory effects. Therefore, future research ought to include an introductory e-visit dedicated to practising sampling methods, allowing participants to become more experienced in the collection techniques. The targeted analysis of the gut microbiota provided an abundance of a panel of 108 gut microbial species, of which 83 were included in the analyses. These microbial taxa are representative of the majority of the human gut microbiome [83]. However, other bacteria may have changed during the interventions and were not covered by the targeted panel. The participants included in the study were all young and healthy females (except for CeD), and the results cannot be generalised to the general population. The study employed a double-blind design; however, it is possible that some participants identified the products they received, since the benchmark products were already commercially available in grocery stores. The participants’ weight and physical activity levels remained unchanged during the study, which strengthens the probability that the dietary interventions induced the observed changes. We did not measure the soluble and insoluble dietary fibre content of the study products; thus, only the total fibre content was assessed in the study. Moreover, since the experimental and control products differed in their macronutrient composition, it is not possible to definitively attribute the observed glycaemic responses solely to fibre or pseudocereals. Thus, the results may reflect the effect of the whole product. Finally, while the participants were instructed to limit cereal consumption to the study products, the study was not a fully controlled dietary intervention. Consequently, the participants may have consumed fibre from non-cereal sources during the study.

## 6. Conclusions

In conclusion, pseudocereals can be used to increase the fibre content of gluten-free products in women with CeD without increasing gastrointestinal symptoms. However, more studies are needed to determine the types and amounts of pseudocereals that may confer health benefits, including improved glycaemic regulation, in individuals with CeD. Whether potential health effects of pseudocereals are exerted through alterations in the gut microbiota needs to be further investigated.

## Figures and Tables

**Table 1:** Participant characteristics (n = 30).

	All Participants	Control Group	Experimental Group	*p*-Value ^5^
Total participants (n)	30	14	16	
Age (years)	31 (25.3–42.5)	38 (25.8–45.5)	29 (23.8–39.5)	0.26
Age at diagnosis (years)	26.5 (13.5–38.5) ^2^	27 (12.0–43.0) ^3^	22.5 (14.3–35.5) ^4^	0.60
Duration of gluten-free diet (years)	8.5 (3.0–13.0) ^2^	9.5 (2.0–13.0) ^3^	8 (3.8–14.8) ^4^	0.70
BMI (kg/m^2^)	22.4 (20.8–24.3)	22.3 (20.8–25.0)	22.6 (20.8–24.2)	0.85
Fasting glucose (mmol/L)	5.3 (5.0–5.6)	5.4 (5.2–5.6) ^1^	5.2 (5.0–5.6)	0.15
C-peptide (pmol/L)	299.4 (265.7–352.4)	286.4 (247.9–350.2)	314.8 (269.9–353.6)	0.45
Total cholesterol (mmol/L)	4.8 (4.3–5.3)	4.9 (4.6–5.4)	4.7 (4.2–5.3)	0.51
HbA1c (mmol/mol)	36.7 (35.5–37.9)	36.6 (35.6–37.5)	36.7 (35.1–38.6)	0.95

^1^ n = 13. ^2^ n = 24. ^3^ n = 10. ^4^ n = 14. Data are given as median (25th–75th percentiles). ^5^ *p*-values ≤ 0.05 indicate statistically significant differences between groups and are calculated by the Mann–Whitney U test. Abbreviations: BMI: Body Mass Index; C-peptide: Connecting peptide; HbA1c: Haemoglobin A1c.

**Table 2:** Daily dietary intake assessed by food frequency questionnaire (FFQ) at screening (n = 30).

	All Participants (n = 30)	Control Group (n = 14)	Experimental Group (n = 16)	*p*-Value ^1^
Energy (kcal)	2164 (1936–2812)	2084 (1957–3138)	2274 (1812–2806)	0.90
Carbohydrate (E%)	41.4 (36.3–45.3)	43.1 (38.3–47.0)	38.6 (35.6–44.5)	0.24
Protein (E%)	16.8 (16.1–18.6)	16.4 (15.9–17.3)	17.4 (16.1–19.8)	0.16
Fat (E%)	36.9 (32.4–40.7)	35.1 (31.9–41.5)	38.0 (32.8–40.8)	0.59
Saturated fat (E%)	12.9 (11.0–14.9)	12.8 (11.1–16.5)	13.0 (10.9–14.8)	0.71
Polyunsaturated fat (E%)	5.9 (5.0–6.8)	5.4 (4.7–6.8)	6.1 (5.4–7.0)	0.21
Alcohol (E%)	1.9 (0.6–3.5)	1.9 (0.6–4.1)	1.9 (0.7–3.3)	0.87
Whole grain (g)	69.0 (43.7–90.1)	67.7 (39.9–74.2)	73.2 (46.1–118.4)	0.38
Dietary fibre (g)	30.5 (24.5–40.7)	25.5 (22.8–42.1)	33.1 (24.9–39.9)	0.35

Data are given as median (25th–75th percentiles). ^1^ *p*-values ≤ 0.05 indicate statistically significant differences between groups and are calculated by the Mann–Whitney U test. Abbreviations: E%: Energy percent; kcal: kilocalorie.

**Table 3:** Dietary fibre sources assessed by food frequency questionnaire (FFQ) at screening (n = 30).

Grams per Day	All Participants (n = 30)	Control Group (n = 14)	Experimental Group (n = 16)	*p*-Value ^1^
Bread, total	120.3 (82.1–174.9)	149.6 (102.4–174.9)	105.8 (59.3–193.0)	0.28
White bread	7.6 (1.6–15.5)	8.7 (3.3–19.0)	6.6 (1.6–9.3)	0.48
Brown bread (<50% whole grain flour)	60.0 (15.0–120.0)	80.0 (15.0–160.0)	40.0 (5.0–80.0)	0.12
Wholegrain bread (>50% whole grain flour)	0.0 (0.0–45.0)	0.0 (0.0–45.0)	0.0 (0.0–39.5)	0.94
Crispbread and flatbread	26.0 (13.0–39.8)	20.0 (0.0–29.3)	32.5 (13.3–49.5)	0.11
Cereal products	142.9 (78.8–193.6)	159.7 (78.8–194.7)	124.2 (70.0–194.6)	0.73
Flour, rice, pasta	58.3 (41.1–104.8)	47.1 (41.7–134.8)	86.3 (40.2–94.3)	0.60
Wholegrain, grains, flour	1.9 (0.9–4.0)	2.4 (1.4–5.0)	1.5 (0.6–2.4)	0.19
Breakfast cereal	9.1 (0.0–24.1)	7.9 (0.0–24.2)	10.1 (0.0–26.0)	0.92
Potatoes	34.3 (15.9–55.8)	31.3 (17.6–68.6)	35.1 (15.2–49.2)	0.59
Vegetables	443.3 (272.5–577.2)	379.2 (203.8–510.0)	479.0 (313.2–583.8)	0.16
Carrots	31.3 (12.2–62.6)	27.9 (11.3–70.5)	31.3 (13.5–60.9)	0.92
Swede	0.0 (0.0–2.5)	0.0 (0.0–2.1)	0.0 (0.0–0.4)	0.44
Cabbage	0.0 (0.0–9.1)	0.0 (0.0–1.8)	0.0 (0.0–4.2)	0.52
Cauliflower	4.9 (0.0–9.1)	5.8 (0.0–16.8)	4.2 (0.0–8.3)	0.55
Broccoli	13.9 (3.4–31.4)	15.8 (1.1–35.6)	13.9 (4.3–28.0)	0.92
Leeks and onions	7.6 (1.7–11.8)	7.0 (0.9–13.4)	8.2 (1.8–11.7)	0.66
Salads and Napa cabbage	19.9 (13.2–29.7)	21.3 (13.9–30.9)	18.3 (12.5–28.7)	0.59
Tomatoes	51.9 (30.9–93.5)	43.4 (13.8–70.1)	65.6 (35.0–111.9)	0.11
Paprika and chili	12.8 (3.0–36.4)	11.2 (0.9–29.7)	22.5 (10.4–38.9)	0.12
Fruit and berries	265.8 (131.8–336.2)	270.5 (147.7–338.1)	265.8 (100.6–345.1)	0.80
Peas and beans	17.5 (3.1–37.1)	12.4 (0.0–30.9)	20.6 (3.1–37.1)	0.44
Citrus fruits	12.0 (1.7–26.9)	8.1 (0.0–27.3)	16.3 (2.2–28.8)	0.53
Apples and pears	48.4 (9.1–100.6)	75.8 (11.8–106.5)	33.5 (9.0–91.9)	0.66

Data are given as median (25th–75th percentiles). ^1^ *p*-values ≤ 0.05 indicate statistically significant differences between groups and are calculated by the Mann–Whitney U test.

**Table 4:** Energy and nutritional content of the study products.

	Control Products				Experimental Products			
	Oatbread (per 100 g)	Grain Bread (per 100 g)	Corn Flakes (per 100 g)	Daily Intake ^1^	Bread (per 100 g)	Bread Rolls (per 100 g)	Muesli (per 100 g)	Daily Intake ^1^
Energy (kcal)	249	273	374	773	228	298	462	882
Protein (g)	2.5	2.3	7.1	9.3	8.4	11.9	18.5	33.9
Fat (g)	5.0	7.3	0.5	13.2	7.3	17.0	27.6	39.6
Saturated fat (g)	0.6	0.8	0.1	1.5	1.0	2.4	3.9	5.6
Monounsaturated fatty acid (g)	2.6	3.3	0.1	6.2	2.3	5.4	8.6	12.5
Polyunsaturated fatty acids (g)	1.5	2.9	0.3	4.8	3.6	8.3	13.8	19.6
Omega-6 (g)	1.0	1.9	0.2	3.2	2.5	6.4	10.3	14.4
Omega-3 (g)	0.5	1.0	0.0	1.6	1.1	1.9	3.4	5.2
Carbohydrates (g)	45.4	47.0	82.1	146.3	28.6	21.0	29.3	85.3
Dietary fibre, total (g)	5.3	4.3	3.7	12.3	6.8	7.5	12.4	24.8
Inulin/FOS (%)	<0.2	<0.2	<0.2	<0.5	<0.2	<0.2	<0.2	<0.6
Fructose (g)	0.1	1.0	0.7	1.8	0.8	<0.04	<0.04	<1.6
Glucose (g)	3.0	3.8	0.7	7.5	1.0	<0.04	<0.04	<2.0
Lactose (g)	<0.04	<0.04	<0.04	<0.1	<0.04	<0.04	<0.04	<0.1
Maltose (g)	<0.04	<0.04	<0.04	<0.1	0.4	<0.0	<0.0	<0.7
Sucrose (g)	<0.04	0.05	5.3	<3.3	0.1	0.7	1.3	1.4
Galactose (g)	<0.04	<0.04	<0.04	<0.1	<0.04	<0.04	<0.04	<0.1
Maltitol (g)	<0.04	<0.04	<0.04	<0.1	<0.04	<0.04	<0.04	<0.1
Sorbitol (g)	<0.04	<0.04	<0.04	<0.1	<0.04	<0.04	<0.04	<0.1
Xylitol (g)	<0.04	<0.04	<0.04	<0.1	<0.04	<0.04	<0.04	<0.1
Mannitol (g)	<0.04	<0.04	<0.04	<0.1	<0.04	<0.04	<0.04	<0.1
Sugar, total (g)	3.1	4.9	6.6	12.3	2.3	0.7	1.3	5.6
Salt (g)	1.1	0.7	1.6	2.7	0.7	0.9	2.5	3.3
Sodium (Na) (mg)	450.0	270.0	620.0	1092.0	280.0	340.0	1000.0	1330.0
Magnesium (Mg) (mg)	17.0	29.0	11.0	52.6	100.0	120.0	210.0	386.0
Iron (Fe) (mg)	2.1	1.3	0.50	3.7	2.3	2.7	5.0	9.0
Selenium (Se) (µg)	5	5	10	10	5	5	7	20
Zinc (Zn) (mg)	0.4	0.6	0.2	1.1	1.9	2.2	3.9	7.2

^1^ Daily minimum intake of test products (control products: six slices of bread (approx. 35 g each slice) and one portion of cornflakes (60 g). Experimental products: four slices of bread (approx. 50 g each slice), one roll (approx. 50 g), and one portion of muesli (60 g)). Abbreviations: g: gram; kcal: kilocalorie; mg: milligram. Analyses conducted by Eurofins AS.

**Table 5:** Effect of control- and experimental products on gut symptoms, physical activity, metabolic and inflammatory markers, and satiety hormone before and after intervention.

	Control Group				Experimental Group				
	Before	After	Change	*p*-Value ^1^	Before	After	Change	*p*-Value ^1^	*p*-Value ^2^
Shannon index	3.24 (3.06–3.51)	3.05 (2.69–3.27)	−0.22 (−0.56–0.20)	0.18	3.00 (2.41–3.37)	3.09 (2.74–3.50)	0.35 (−0.47–0.67)	0.60	0.212
Bristol Stool Chart	2.69 (2.00–3.25)	2.75 (2.00–4.25)	0.00 (−0.50–0.63)	0.57	3.00 (2.00–3.38)	3.00 (2.25–3.88)	0.50 (0.00–1.00)	0.23	0.36
GSRS-IBS Pain	4.83 (3.00–6.00)	3.50 (2.00–6.25)	−0.50 (−2.17–0.25)	0.28	3.00 (2.00–5.00)	3.00 (2.00–6.00)	0.00 (0.00–2.00)	0.40	0.13
GSRS-IBS Bloating	7.96 (6.50–10.00)	7.25 (5.00–9.00)	−0.50 (−2.94–1.25)	0.41	7.25 (6.00–9.38)	8.00 (5.50–10.75)	1.50 (−0.75–2.75)	0.16	0.12
GSRS-IBS Constipation	4.42 (2.00–7.25)	4.00 (2.00–6.00)	−0.92 (−2.00–0.00)	0.25	3.50 (2.00–5.75)	2.50 (2.00–5.50)	0.00 (−2.75–0.75)	0.36	0.53
GSRS-IBS Diarrhoea	8.42 (5.00–10.50)	6.00 (4.00–9.00)	−2.00 (−3.67–0.13)	**0.01**	6.00 (4.25–8.00)	5.75 (4.25–8.00)	0.00 (−1.00–1.00)	0.87	**0.04**
GSRS-IBS Early satiety	4.92 (3.75–6.00)	4.50 (3.00–6.25)	0.00 (−1.46–1.00)	0.96	4.50 (3.00–6.00)	5.00 (3.25–7.00)	0.00 (−1.00–1.75)	0.53	0.57
GSRS-IBS Overall score	33.50 (21.50–36.25)	25.25 (19.75–35.38)	−2.83 (−10.67)	0.12	27.00 (17.50–34.75)	24.00 (20.25–36.75)	3.00 (−3.75–5.75)	0.50	0.12
IPAQ PA category	2.00 (2.00–2.00)	2.00 (1.00–2.25)	0.00 (−1.00–0.00)	0.26	2.00 (1.25–2.00)	2.00 (2.00–2.00)	0.00 (0.00–0.75)	0.53	0.25
Total cholesterol (mmol/L)	4.93 (4.56–5.40)	5.25 (4.43–5.54)	−0.09 (−0.49–1.03)	0.83	4.68 (4.25–5.25)	4.55 (3.89–5.75)	0.12 (−0.82–0.99)	1.00	0.74
C-peptide (pmol/L)	286.42 (247.92–350.23)	307.05 (240.45–340.56)	3.22 (−67.85–65.32)	0.98	314.83 (269.88–353.62)	262.61 (210.47–393.58)	−27.62 (−84.35–67.00)	0.47	0.53
HbA1c (mmol/mol)	36.64 (35.64–37.53)	36.90 (35.58–38.86)	0.23 (−0.71–2.62)	0.25	36.72 (35.09–38.64)	35.37 (35.11–37.65)	−0.47 (−1.35–0.49)	0.23	0.09
CRP (mg/L)	0.79 (0.12–1.51)	0.45 (0.14–0.91)	0.03 (−0.42–0.18)	0.88	0.40 (0.22–0.69)	0.69 (0.40–1.94)	0.14 (−0.02–0.97)	0.09	0.14
PYY (ng/mL)	0.23 (0.19–0.28)	0.22 (0.18–0.29)	−13.17 (−74.38–61.79)	0.83	0.26 (0.19–0.33)	0.28 (0.22–0.31)	9.97 (−49.23–42.72)	0.72	0.87
IL-1β (pg/mL)	4.63 (2.40–13.43)	5.11 (1.35–12.55)	−1.31 (−8.89–3.85)	0.33	4.88 (1.11–13.04)	5.21 (0.77–11.15)	−0.22 (−6.55–4.52)	0.92	0.51
IL-1Ra (ng/mL)	2.32 (1.31–3.12)	2.75 (2.10–3.52)	433.82 (−218.32–991.12)	0.07	2.55 (0.32–3.50)	3.34 (2.26–3.74)	597.29 (−1051.16–2650.54)	0.15	0.87
IL-8 (pg/mL)	49.15 (42.76–54.03)	46.14 (41.55–56.51)	−2.32 (−11.04–4.89)	0.43	48.02 (40.45–58.61)	56.93 (42.28–75.67)	−0.92 (−6.96–26.89)	0.38	0.41
MCP-1 (ng/mL)	0.35 (0.29–0.42)	0.35 (0.30–0.41)	−7.28 (−49.42–36.82)	0.98	0.35 (0.27–0.43)	0.33 (0.29–0.41)	10.07 (−59.14–53.92)	0.92	1.00

Data are given as median (25th–75th percentiles). *p*-values ≤ 0.05 indicate significant differences within or between groups. Significant differences within and between groups are marked in bold. ^1^ Differences within groups were analysed by Wilcoxon signed-rank test ^2^ Differences in change between groups were analysed by Mann–Whitney U test. Abbreviations: GSRS-IBS: Gastrointestinal Symptom Rating Scale-Irritable Bowel Syndrome; CRP: C-Reactive Protein; IL: Interleukin; IPAQ: International Physical Activity Questionnaire; MCP-1: Monocyte Chemoattractant Protein-1; PA: Physical Activity; PYY: Peptide YY.

**Table 6:** The abundance of gut microbiota in control group and experimental group before and after intervention.

Species		Control Group					Experimental Group				EG/ CG		*p*-Value ^2^	Q-Value
	Before	After	After/ Before		*p*-Value ^1^	Before	After	After/ Before		*p*-Value ^1^				
	Mean (SD)	Mean (SD)	Log2 Ratio	95% CI		Mean (SD)	Mean (SD)	Log2 Ratio	95% CI		Log2 Ratio	95% CI		
*Anaerostipes hadrus*	9.14 (1.15)	8.07 (1.24)	−1.07	−2.02, −0.11	**0.03**	8.56 (0.69)	8.75 (0.65)	0.19	−0.38, 0.76	0.48	1.26	−2.33, −0.18	**0.02**	0.69
*Bacteroides caccae*	13.08 (0.76)	11.65 (2.34)	−1.43	−2.64, −0.22	**0.02**	12.33 (1.13)	12.38 (1.56)	0.04	−0.82, 0.91	0.91	1.47	−2.91, −0.37	**0.04**	0.69
*Candida* (genus)	8.82 (1.31)	7.93 (1.05)	−0.89	−1.64, −0.14	**0.02**	7.87 (1.82)	7.65 (2.05)	−0.22	−1.39, 0.95	0.69	0.67	−1.97, 0.63	0.30	0.78
*Coprococcus catus*	9.57 (0.80)	7.76 (2.78)	−1.81	−3.39, −0.22	**0.03**	8.87 (1.00)	7.92 (3.52)	−0.95	−3.14, 1.24	0.36	0.85	−3.39, 1.68	0.50	0.88
*Dorea longicatena*	11.07 (0.74)	10.38 (0.68)	−0.70	−1.33, −0.06	**0.03**	10.46 (0.70)	10.59 (0.52)	0.13	−0.28, 0.53	0.50	0.83	−1.55, −0.10	**0.03**	0.69
*Escherichia coli*	10.91 (1.72)	9.24 (1.80)	−1.67	−3.19, −0.14	**0.03**	9.23 (2.73)	10.22 (2.90)	0.99	−0.89, 2.88	0.27	2.66	−4.94, −0.34	**0.02**	0.69
*Faecalibacterium prausnitzii A2-165*	13.96 (0.61)	12.82 (1.69)	−1.14	−2.06, −0.22	**0.02**	13.02 (1.67)	13.05 (1.76)	0.04	−0.74, 0.81	0.92	1.17	−2.33, −0.2	**0.05**	0.69
*Phascolarctobacterium faecium*	12.00 (0.56)	10.59 (2.07)	−1.41	−2.51, −0.32	**0.02**	10.22 (3.59)	10.20 (2.81)	−0.02	−2.03, 1.99	0.98	1.39	−3.52, 0.73	0.19	0.69
*Ruminococcus lactaris*	10.78 (0.95)	9.58 (2.10)	−1.20	−2.27, −0.14	**0.03**	10.34 (2.17)	10.17 (1.69)	−0.18	−1.22, 0.87	0.72	1.03	−2.44, 0.39	0.15	0.69

Values are Log2-transformed and presented as mean (SD). Differences within groups and between groups are presented as Log2 ratio (after/before) and Log2 ratio (experimental group/control group). Significant differences (*p* ≤ 0.05) within and between groups are marked in bold. ^1^ Differences within groups were analysed by paired *t*-test. ^2^ Differences in change between groups were analysed by independent *t*-test. Abbreviations: CG: Control Group; EG: Experimental Group; SD: Standard Deviation. Q-values represent the final significance Benjamini–Hochberg False discovery Rate (FDR) correction across the 83 targeted bacterial taxa.

## Data Availability

The datasets presented in this article are not readily available because of privacy restrictions. Requests to access the datasets should be directed to corresponding author.

## Supplementary Materials

The following supporting information can be downloaded at: https://www.mdpi.com/article/10.3390/nu18213462/s1, Table S1: The abundance of gut microbiota in control group and experimental group before and after intervention.

## Abbreviations

CRF	A Case Report Form
BSC	Bristol Stool Chart
CeD	Coeliac disease
CRP	C-reactive Protein
DGV	Det Glutenfrie Verkstedet, Norway
DBS	Dried Blood Spots
ECL	Electrochemiluminescence
ELISA	Enzyme-linked Immunosorbent Assay
EFSA	European Food Safety Authority
FFQ	Food Frequency Questionnaire
GC-FID	Gas Chromatography–Flame Ionisation Detection
GSRS-IBS	Gastrointestinal Symptoms Rating Scale-Irritable Bowel Syndrome
HbA1c	Haemoglobin A1c
IFN-Y	Interferon-gamma
IL	Interleukin
LLOQ	Lower Limit of Quantitation
MCP-1	Monocyte Chemoattractant Protein-1
OGTT	Oral Glucose Tolerance Test
PYY	Peptide YY
RPM	Revolutions Per Minute
TNF-α	Tumor Necrosis Factor Alpha
