# Supplementary material for: The Effect of Fibre-Rich Gluten Free Products on Glycaemic Regulation, Gut Symptoms, and Targeted Gut Microbial Taxa in Women with Coeliac Disease: A Randomised Controlled Trial
Source: Nutrients. 2026 Oct 22;18(21):3462. doi: 10.3390/nu18213462 (PMC13765387; doi:10.3390/nu18213462)
Supplement: Supplementary file 1 [file nutrients-18-03462-s001.zip › nutrients-4598782-supplementary.pdf]

Supplementary Table S1. The abundance of gut microbiota in control group and experimental group before and after intervention.

	Control group					Experimental group								
Species	Before	After	After/ before		P-value[1]	Before	After	After/ before		P-value[1]	EG/CG		P-value[2]	Q-value
	Mean (SD)	Mean (SD)	Log2 ratio	95% CI		Mean (SD)	Mean (SD)	Log2 ratio	95% CI		Log2 ratio	95% CI		
Akkermansia muciniphila	8.79 (2.43)	8.58 (3.67)	-0.21	-2.38, 1.96	0.84	9.27 (2.04)	9.01 (1.39)	-0.26	-1.71, 1.20	0.71	-0.05	-2.48, 2.57	0.97	1.00
Akkermansia muciniphila Aml	10.19 (1.59)	9.14 (3.27)	-1.04	-2.89, 0.80	0.24	9.30 (2.21)	10.09 (2.01)	0.79	-1.17, 2.74	0.40	1.83	-4.38, 0.72	0.15	0.69
Alistipes communis	13.39 (0.64)	9.54 (1.77)	-0.85	-1.85, 0.16	0.09	9.82 (1.66)	9.90 (1.39)	0.07	-0.89, 1.04	0.88	0.92	-2.25, 0.41	0.17	0.69
Alistipes finegoldii	6.24 (3.61)	3.48 (4.21)	-2.77	-5.57, 0.03	0.05	6.08 (4.49)	6.39 (3.74)	0.31	-2.28, 2.89	0.80	3.07	-6.70, 0.55	0.09	0.69
Alistipes onderdonkii	10.96 (1.92)	10.04 (3.32)	-0.91	-2.97, 1.14	0.36	11.70 (2.04)	11.42 (2.08)	-0.28	-1.30, 0.74	0.56	0.63	-2.87, 1.60	0.57	0.89
Alistipes putredinis	13.45 (1.94)	12.40 (4.02)	-1.05	-3.13, 1.03	0.30	13.17 (1.46)	12.17 (3.90)	-1.00	-3.14, 1.14	0.33	0.05	-2.89, 2.79	0.97	1.00
Alistipes senegalensis	6.74 (3.79)	5.96 (3.98)	-0.78	-3.70, 2.15	0.58	6.05 (4.30)	7.19 (3.39)	1.15	-1.39, 3.68	0.34	1.93	-5.63, 1.78	0.29	0.78
Alistipes shahii	10.34 (1.52)	10.03 (3.12)	-0.31	-2.24, 1.63	0.74	11.26 (1.06)	10.64 (1.65)	-0.62	-1.43, 0.19	0.12	-0.31	-1.74, 2.37	0.76	0.98
Anaerostipes hadrus	9.14 (1.15)	8.07 (1.24)	-1.07	-2.02, -0.11	**0.03**	8.56 (0.69)	8.75 (0.65)	0.19	-0.38, 0.76	0.48	1.26	-2.33, -0.18	**0.02**	0.69
Bacteroides caccae	13.08 (0.76)	11.65 (2.34)	-1.43	-2.64, -0.22	**0.02**	12.33 (1.13)	12.38 (1.56)	0.04	-0.82, 0.91	0.91	1.47	-2.91, -0.37	**0.04**	0.69
Bacteroides cellulosilyticus	12.85 (2.07)	10.55 (5.17)	-2.30	-5.52, 0.91	0.15	12.34 (2.64)	11.47 (4.40)	-0.87	-3.43, 1.69	0.47	1.43	-5.38, 2.52	0.46	0.88
Bacteroides coprocola	8.35 (4.92)	6.53 (5.41)	-1.82	-6.57, 2.93	0.42	3.61 (4.82)	6.69 (5.56)	3.09	-1.37, 7.55	0.16	4.91	-11.12, 1.30	0.12	0.69
Bacteroides eggerthii	8.34 (2.00)	5.93 (3.66)	-2.41	-4.90, 0.08	0.06	6.41 (3.35)	6.52 (4.08)	0.11	-2.43, 2.65	0.92	2.52	-5.90, 0.86	0.14	0.69
Bacteroides faecis	9.57 (4.02)	8.07 (2.94)	-1.51	-3.93, 0.92	0.20	8.60 (4.24)	9.25 (2.74)	0.65	-1.80, 3.10	0.58	2.16	-5.43, 1.12	0.19	0.69

Bacteroides finegoldii	7.38 (4.28)	7.15 (4.52)	-0.22	-3.07, 2.63	0.87	3.14 (3.66)	3.98 (3.92)	0.83	-2.03, 3.70	0.54	1.06	-4.90, 2.79	0.58	0.89
Bacteroides fragilis	10.84 (3.24)	10.77 (1.78)	-0.07	-1.67, 1.52	0.92	11.51 (1.63)	11.53 (1.02)	0.02	-0.88, 0.93	0.95	0.10	-1.89, 1.68	0.91	1.00
Bacteroides intestinalis	11.08 (3.88)	9.80 (5.08)	-1.28	-4.60, 2.03	0.42	5.76 (5.05)	7.93 (5.69)	2.17	-3.00, 7.33	0.38	3.45	-9.18, 2.28	0.23	0.72
Bacteroides nordii	6.43 (3.69)	4.74 (3.81)	-1.69	-4.23, 0.86	0.18	4.43 (4.00)	4.09 (4.00)	-0.34	-1.86, 1.17	0.63	1.35	-4.21, 1.53	0.34	0.83
Bacteroides ovatus	12.45 (1.86)	11.49 (3.58)	-0.95	-3.22, 1.31	0.38	12.87 (2.02)	11.88 (3.84)	-0.99	-3.36, 1.39	0.38	-0.03	-3.08, 3.15	0.98	1.00
Bacteroides plebeius	6.85 (5.48)	5.46 (5.40)	-1.39	-6.05, 3.28	0.53	3.97 (4.86)	5.18 (5.23)	1.21	-2.50, 4.91	0.49	2.60	-8.31, 3.12	0.36	0.83
Bacteroides stercoris	9.59 (3.60)	8.38 (3.36)	-1.21	-3.38, 1.38	0.33	6.15 (5.30)	7.53 (3.90)	1.38	-2.32, 5.09	0.43	2.59	-6.82, 1.64	0.22	0.72
Bacteroides thetaiotaomicron	12.81 (0.88)	11.76 (3.91)	-1.05	-3.27, 1.17	0.33	12.30 (1.85)	11.25 (3.55)	-1.04	-3.05, 0.96	0.28	0.00	-2.86, 2.85	1.00	1.00
Bacteroides uniformis	15.27 (0.97)	14.14 (4.17)	-1.13	-3.52, 1.26	0.33	15.94 (1.11)	13.99 (4.25)	-1.95	-4.47, 0.57	0.12	-0.82	-2.48, 4.12	0.61	0.92
Bacteroides xylanisolvens	12.78 (1.25)	12.65 (2.45)	-0.13	-1.54, 1.28	0.84	12.25 (2.44)	12.42 (1.38)	0.16	-1.01, 1.34	0.77	0.29	-2.05, 1.46	0.73	0.96
Barnsiella intestinihominis	11.35 (1.10)	9.45 (4.12)	-1.86	-4.35, 0.63	0.13	11.48 (1.45	11.10 (1.44)	-0.38	-1.32, 0.57	0.40	1.48	-4.09, 1.13	0.25	0.72
Bifidobacterium adolescentis	4.41 (4.11)	3.60 (4.38)	-0.81	-4.51, 2.88	0.64	7.57 (3.09)	6.97 (3.73)	-0.60	-3.38, 2.18	0.65	0.21	-4.67, 4.24	0.92	1.00
Bifidobacterium longum	4.76 (3.64)	5.36 (3.38)	0.60	-2.60, 3.81	0.69	5.56 (2.80)	4.78 (3.13)	-0.78	-3.40, 1.84	0.53	-1.38	-2.58, 5.35	0.48	0.88
Bifidobacterium longum subsp. longum	3.32 (4.25)	4.19 (4.38)	0.87	-2.87, 4.62	0.62	6.31 (3.09)	3.98 (4.01)	-2.33	-5.14, 0.48	0.10	-3.20	-1.30, 7.71	0.16	0.69
Bilophila wadsworthia	8.19 (1.72)	7.53 (3.34)	-0.65	-2.75, 1.45	0.51	9.27 (1.96)	8.46 (2.84)	-0.81	-2.80, 1.19	0.40	-0.16	-2.60, 2.91	0.91	1.00
Blautia obeum	10.07 (1.17)	9.88 (3.00)	-0.20	-1.83, 1.44	0.80	9.91 (0.77)	8.42 (3.80)	-1.50	-3.96, 0.96	0.21	-1.30	-1.46, 4.07	0.34	0.83
Blautia wexlerae	10.73 (1.05)	9.26 (2.86)	-1.47	-3.19, 0.24	0.09	10.35 (1.35)	10.49 (1.57)	0.14	-0.61, 0.90	0.68	1.62	-3.45, 0.21	0.08	0.69
Candida (genus)	8.82 (1.31)	7.93 (1.05)	-0.89	-1.64, -0.14	**0.02**	7.87 (1.82)	7.65 (2.05)	-0.22	-1.39, 0.95	0.69	0.67	-1.97, 0.63	0.30	0.78
Clostridium bolteae	7.03 (3.07)	5.66 (3.77)	-1.36	-4.37, 1.64	0.35	6.80 (3.11)	7.39 (2.31)	0.58	-0.99, 2.15	0.43	1.95	-5.23, 1.33	0.24	0.72
Clostridium leptum	7.98 (1.39)	7.05 (2.22)	-0.93	-2.07, 0.22	0.10	6.83 (2.22)	6.26 (2.62)	-0.57	-2.47, 1.33	0.53	0.36	-2.43, 1.71	0.72	0.96

Collinsella aerofaciens	9.59 (3.73)	9.54 (4.51)	-0.05	-3.32, 3.22	0.97	10.42 (2.17)	8.76 (4.10)	-1.65	-3.77, 0.46	0.11	-1.60	-2.17, 5.37	0.39	0.83
Coprococcus catus	9.57 (0.80)	7.76 (2.78)	-1.81	-3.39, -0.22	0.03	8.87 (1.00)	7.92 (3.52)	-0.95	-3.14, 1.24	0.36	0.85	-3.39, 1.68	0.50	0.88
Coprococcus comes	10.23 (0.82)	9.29 (2.76)	-0.94	-2.54, 0.66	0.23	9.86 (0.95)	8.95 (2.78)	-0.91	-2.63, 0.81	0.27	0.03	-2.26, 2.19	0.98	1.00
Dialister invisus	9.37 (1.05)	8.79 (3.29)	-0.58	-2.50, 1.34	0.53	9.20 (1.78)	9.06 (1.57)	-0.14	-1.17, 0.88	0.77	0.44	-2.56, 1.68	0.67	0.94
Dorea formicigenerans	8.58 (2.71)	8.64 (0.88)	0.06	-1.48, 1.60	0.93	9.07 (0.67)	9.16 (0.70)	0.09	-0.26, 0.43	0.59	0.03	-1.59, 1.53	0.97	1.00
Dorea longicatena	11.07 (0.74)	10.38 (0.68)	-0.70	-1.33, -0.06	**0.03**	10.46 (0.70)	10.59 (0.52)	0.13	-0.28, 0.53	0.50	0.83	-1.55, -0.10	**0.03**	0.69
Escherichia coli	10.91 (1.72)	9.24 (1.80)	-1.67	-3.19, -0.14	**0.03**	9.23 (2.73)	10.22 (2.90)	0.99	-0.89, 2.88	0.27	2.66	-4.94, -0.34	**0.02**	0.69
Eubacterium eligens	11.78 (1.27)	10.48 (3.15)	-1.30	-3.30, 0.70	0.18	11.84 (1.53)	10.83 (3.36)	-1.01	-3.29, 1.27	0.35	0.29	-3.16, 2.58	0.84	0.98
Eubacterium rectale	14.30 (1.04)	13.87 (1.34)	-0.44	-1.40, 0.53	0.35	13.44 (0.97)	13.75 (0.63)	0.31	-0.39, 1.01	0.35	0.75	-1.90, 0.40	0.19	0.69
Eubacterium siraeum	11.38 (1.88)	9.46 (3.32)	-1.92	-4.17, 0.33	0.09	9.14 (4.07)	8.74 (5.56)	-0.40	-3.34, 2.54	0.77	1.52	-5.00, 1.96	0.38	0.83
Eubacterium ventriosum	11.27 (0.87)	10.32 (3.20)	-0.95	-2.87, 0.98	0.31	10.74 (1.39)	9.31 (4.17)	-1.42	-3.55, 0.70	0.17	-0.47	-2.25, 3.19	0.72	0.96
Faecalibacterium cf. prausnitzii KLE1255	11.61 (1.49)	10.97 (1.17)	-0.64	-1.50, 0.22	0.13	12.66 (3.67)	11.28 (1.93)	-1.40	-3.27, 0.49	0.13	-0.76	-1.15, 2.66	0.42	0.87
Faecalibacterium prausnitzii	10.98 (1.52)	10.06 (3.32)	-0.93	-2.81, 0.96	0.31	10.87 (1.94)	9.52 (3.39)	-1.35	-2.97, 0.28	0.10	-0.43	-1.96, 2.81	0.72	0.96
Faecalibacterium prausnitzii A2-165	13.96 (0.61)	12.82 (1.69)	-1.14	-2.06, -0.22	**0.02**	13.02 (1.67)	13.05 (1.76)	0.04	-0.74, 0.81	0.92	1.17	-2.33, -0.2	**0.05**	0.69
Faecalibacterium prausnitzii AHMP21	7.69 (5.30)	5.42 (3.78)	-2.27	-5.15, 0.61	0.11	6.48 (4.70)	5.62 (4.82)	-0.86	-4.38, 2.66	0.61	1.41	-5.70, 2.88	0.51	0.88
Faecalibacterium prausnitzii CNCM4575	9.68 (4.17)	9.62 (4.39)	-0.07	-3.70, 3.57	0.97	6.02 (5.92)	7.73 (5.55)	1.71	-2.99, 6.41	0.44	1.77	-7.36, 3.82	0.52	0.88
Faecalibacterium prausnitzii L2-6	13.58 (0.77)	13.45 (0.40)	-0.13	-0.54, 0.28	0.50	13.06 (1.48)	13.63 (0.76)	0.56	-0.20, 1.33	0.13	0.69	-1.50, 0.11	0.09	0.69
Faecalibacterium prausnitzii M21/2	13.14 (0.96)	11.82 (3.76)	-1.32	-3.52, 0.89	0.22	12.58 (2.18)	11.69 (3.65)	-0.89	-3.70, 1.91	0.50	0.43	-3.79, 2.93	0.80	0.98
Flavonifractor plautii	8.50 (0.79)	8.08 (1.01)	-0.42	-1.05, 0.21	0.17	8.06 (0.98)	8.22 (0.59)	0.16	-0.28, 0.59	0.46	0.58	-1.32, 0.16	0.12	0.69

Glemmiger formicilis	11.07 (0.89)	10.48 (1.33)	-0.59	-1.37, 0.19	0.13	10.65 (1.38)	10.78 (0.77)	0.13	-0.63, 0.89	0.72	0.72	-1.75, 0.31	0.16	0.69
Gordonibacter pamelaeae	1.81 (2.53)	2.06 (2.91)	0.25	-1.54, 2.04	0.77	3.53 (2.67)	4.13 (2.57)	0.59	-1.82, 3.00	0.60	0.34	-3.17, 2.48	0.80	0.98
Haemophilus parainfluenzae	8.48 (1.40)	7.57 (2.80)	-0.91	-2.60, 0.78	0.26	7.30 (2.0)	7.05 (3.14)	-0.25	-2.02, 1.52	0.76	0.66	-2.98, 1.66	0.56	0.89
Holdemanella biformis	7.97 (3.10)	6.91 (3.83)	-1.07	-3.53, 1.39	0.37	3.80 (2.99)	5.04 (3.73)	1.24	-1.81, 4.29	0.39	2.31	-6.00, 1.38	0.21	0.72
Intestinibacter bartletti	4.41 (3.02)	4.27 (2.57)	-0.14	-2.20, 1.92	0.89	5.05 (2.24)	5.19 (2.72)	0.13	-1.54, 1.81	0.87	0.27	-2.82, 2.23	0.83	0.98
Methanobrevibacter smithii	7.98 (2.81)	6.23 (3.88)	-1.75	-4.72, 1.22	0.23	4.51 (4.81)	6.24 (4.59)	1.73	-1.49, 4.96	0.26	3.48	-7.64, 0.67	0.10	0.69
Odoribacter splanchnicus	11.42 (0.72)	10.50 (3.06)	-0.93	-2.73, 0.87	0.29	11.07 (1.37)	10.16 (3.14)	-0.92	-2.95, 1.12	0.35	0.01	-2.58, 2.56	0.99	1.00
Parabacteroides distasonis	12.98 (1.20)	12.00 (3.60)	-0.98	-3.04, 1.09	0.32	12.41 (1.31)	12.36 (1.06)	-0.04	-0.89, 0.81	0.92	0.94	-3.12, 1.25	0.39	0.83
Parabacteroides goldsteinii	5.74 (4.00)	5.76 (3.99)	0.02	-2.32, 2.37	0.98	4.46 (3.79)	4.88 (4.11)	0.42	-1.98, 2.82	0.71	0.39	-3.58, 2.79	0.80	0.98
Parabacteroides merdae	11.39 (2.36)	9.26 (5.11)	-2.13	-5.34, 1.08	0.18	10.34 (3.89)	9.31 (5.39)	-1.03	-4.80, 2.83	0.57	1.10	-5.84, 3.64	0.64	0.93
Paraprevotella clara	9.67 (4.65)	7.31 (5.43)	-2.36	-6.67, 1.96	0.26	4.78 (5.47)	7.31 (6.06)	2.53	-1.97, 7.03	0.24	4.89	-10.82, 1.03	0.10	0.69
Parasutterella excrementihominis	8.68 (1.28)	8.16 (1.85)	-0.52	-1.69, 0.66	0.36	7.81 (2.62)	8.00 (2.94)	0.19	-1.82, 2.20	0.84	0.71	-2.88, 1.46	0.51	0.88
Phascolarctobacterium faecium	12.00 (0.56)	10.59 (2.07)	-1.41	-2.51, -0.32	**0.02**	10.22 (3.59)	10.20 (2.81)	-0.02	-2.03, 1.99	0.98	1.39	-3.52, 0.73	0.19	0.69
Phocaeicola dorei	14.26 (1.11)	12.39 (4.25)	-1.87	-4.29, 0.55	0.12	13.92 (1.57)	12.84 (4.19)	-1.08	-3.45, 1.29	0.34	0.80	-4.02, 2.43	0.62	0.92
Phocaeicola massiliensis	12.83 (3.05)	12.14 (2.01)	-0.69	-2.36, 0.98	0.39	9.66 (4.09)	9.86 (2.66)	0.21	-2.40, 2.82	0.87	0.90	-3.79, 2.00	0.53	0.88
Phocaeicola vulgatus	15.06 (1.42)	13.67 (4.38)	-1.39	-3.97, 1.20	0.27	14.28 (2.53)	12.40 (5.08)	-1,88	-4.64, 0.88	0.16	-0.50	-3.09, 4.08	0.78	0.98
Prevotella copri	9.50 (6.74)	8.98 (6.61)	-0.52	-5.48, 4.44	0.82	13.04 (2.80)	9.83 (6.05)	-3.21	-7.03, 0.62	0.09	-2.69	-3.34, 8.71	0.37	0.83
Roseburia hominis	11.25 (1.00)	9.31 (4.02)	-1.94	-4.39, 0.50	0.11	11.81 (1.24)	11.22 (1.26)	-0.59	-1.48, 0.30	0.17	1.35	-3.91, 1.20	0.29	0.78
Roseburia intestinalis	10.24 (1.00)	9.23 (2.97)	-1.02	-2.86, 0.82	0.25	9.22 (1.31)	9.63 (3.25)	0.41	-1.43, 2.24	0.64	1.42	-3.89, 1.05	0.25	0.72
Roseburia inulinivorans	12.74 (1.06)	11.64 (3.56)	-1.10	-3.16, 0.97	0.27	12.29 (1.09)	11.13 (3.51)	-1.16	-3.32, 1.00	0.27	-0.06	-2.78, 2.90	0.96	1.00

Ruminococcus bicirculans	10.12 (1.69)	8.37 (3.89)	-1.76	-3.86, 0.35	0.09	9.52 (1.91)	9.60 (3.56)	0.08	-1.42, 1.58	0.91	1.84	-4.33, 0.65	0.14	0.69
Ruminococcus bromii	12.79 (2.02)	11.77 (1.65)	-1.02	-2.25, 0.21	0.10	11.38 (2.07)	11.64 (1.90)	0.26	-1.20, 1.72	0.71	1.28	-3.08, 0.552	0.16	0.69
Ruminococcus gnavus	3.92 (3.60)	2.46 (3.61)	-1.46	-3.63, 0.71	0.17	5.24 (2.72)	3.55 (3.57)	-1.69	-3.91, 0.53	0.12	-0.23	-2.72, 3.18	0.88	1.00
Ruminococcus lactaris	10.78 (0.95)	9.58 (2.10)	-1.20	-2.27, -0.14	**0.03**	10.34 (2.17)	10.17 (1.69)	-0.18	-1.22, 0.87	0.72	1.03	-2.44, 0.39	0.15	0.69
Ruminococcus torques	11.34 (1.29)	10.59 (1.80)	-0.74	-2.07, 0.58	0.25	9.99 (3.31)	9.53 (4.31)	-0.46	-3.12, 2.20	0.72	0.29	-3.04, 2.47	0.83	0.98
Streptococcus thermophilus	8.36 (1.81)	7.14 (3.34)	-1.23	-3.65, 1.19	0.29	7.54 (3.10)	7.50 (3.48)	-0.04	-2.65, 2.57	0.98	1.19	-4.56, 2.18	0.48	0.88
Subdoligranulum sp. (group 2)	10.78 (1.03)	9.94 (2.16)	-0.84	-2.02, 0.34	0.15	9.96 (1.84)	9.68 (3.01	-0.28	-1.46, 0.90	0.61	0.56	-2.14, 1.03	0.48	0.88
Subdoligranulum sp. (group 3)	10.86 (1.39)	10.51 (0.97)	-0.35	-1.02, 0.31	0.27	10.63 (1.10)	10.46 (0.89)	-0.16	-0.76, 0.43	0.56	0.19	-1.04, 0.67	0.66	0.94
Subdoligranulum sp. (group 4)	8.99 (0.86)	8.01 (2.63)	-0.97	-2.68, 0.73	0.24	8.83 (0.78)	8.35 (0.74)	-0.48	-1.05, 0.10	0.10	0.50	-2.26, 1.27	0.57	0.89
Sutturella wadsworthensis	9.21 (1.65)	8.84 (3.54)	-0.36	-2.64, 1.92	0.74	9.79 (2.61)	8.31 (4.04)	-1.48	-3.66, 0.70	0.16	-1.12	-1.90, 4.12	0.45	0.88

Values are Log2-transformed and presented as mean (SD). Differences within groups and between groups are presented as Log2 ratio (after/before) and Log2 ratio (experimental group/control group). Significant differences ($P \leq 0.05$) within and between groups are marked in bold.

[1]Differences within groups were analysed by a paired t-test.

[2]Differences in change between groups were analysed by an independent t-test.

Abbreviations: SD: standard deviation.

Q-values represent the final significance after Benjamini-Hochberg False discovery Rate (FDR) correction across the 83 targeted bacterial taxa.
